# Nanotechnologies in Textiles

**DOI:** 10.3390/ma15041466

**Published:** 2022-02-16

**Authors:** Tomas Tamulevičius

**Affiliations:** 1 Institute of Materials Science, Kaunas University of Technology, K. Baršausko St. 59, LT-51423 Kaunas, Lithuania; tomas.tamulevicius@ktu.lt; 2 Department of Physics, Kaunas University of Technology, Studentų St. 50, LT-51368 Kaunas, Lithuania

## 1. Introduction

Textiles, originally made from natural fiber materials, have thousands of years of history [1]. The advent of synthetic materials has made substantial changes in the textile industry, but recent progress in the development of nanomaterials is promising one more important transformation. The contemporary technologies used for textiles are struggling to meet societal demands and challenges, such as stain repellence, wrinkle-freeness, static elimination, passive cooling, smart wear compatibility, and sufficient electrical conductivity of fibers without compromising comfort and flexibility [2,3]. This inevitably requires the development of new materials along with the associated deposition and/or processing methods. To bridge this gap, nanomaterials, heterogeneous composite materials with embedded nanoparticles [4,5], and 2D materials [6,7] are stepping in and providing the required functionalities and properties that have so far been elusive using other means, including antimicrobial properties, hydrophobic properties, catalytic performance, handling of odors, controlled drug release, and response to external stimuli via electrical, color, or physiological signals without spoiling their durability. The conventional materials used in textiles could be replaced by the nonwoven nanofibrous materials deposited by the electrospinning technique [8], together with their further post-processing for gaining extra functionalities [9]. The electrospinning method enables polymer-based nanofibers ‘loaded’ with different additives including nanoparticles, enzymes, drugs, or catalysts [10]. Such technologies pave the way for the use of nano textiles in a manifold of areas, including biomedical applications [11]. Nanotextiles are already in use at an industrial level. The utilization of nanomaterials also raises the issue of risk factors including nanotoxicity, nanomaterial release during washing, and environmental impact of nanotextiles based on life cycle assessments [12].

## 2. Short Description of the Articles Presented in This Special Issue

The Special Issue “Nanotechnologies in Textiles” published in the journal “Materials” resulted in four peer-reviewed original research articles, and one review article covering current research trends and future perspectives in nanotextiles.

A growing interest in multifunctional textiles exhibiting antimicrobial properties [13,14], UV protection, photocatalytic and self-cleaning properties [15,16], fosters the development of hybrid nanomaterials, their synthesis, and deposition methods. Silver nanoparticles were known for years as antimicrobial material able to fight against severe infections related to antibiotic-resistant bacteria. Juknius et al. [13] have demonstrated a medical patch prototype containing synthetic silk coated with diamond-like carbon nanocomposite thin films doped with 3.4 at.% of silver nanoparticles (DLC:Ag). The microbiological experiments of patch prototype with magnetron sputtered films demonstrated the inactivation of 99% of *S. aureus* bacteria, while a preliminary preclinical study using laboratory animals demonstrated that guinea pigs’ wounds on the skin surface infected with the methicillin-resistant *S. aureus* (MRSA) healed faster compared with the control. Bolskis et al. [14] investigated myrrh extract, an organic alternative exhibiting antimicrobial and antifungal properties, embedded in synthetic thermoplastic biopolymer polylactide (PLA) obtained from renewable resources. Film and melt-spun PLA/myrrh multifilament yarn fiber testing revealed that the PLA granules modified with myrrh extracts (aqueous and ethanolic) caused a decrease in breaking stress and elongation at the break of PLA films. Similarly, fibers of PLA with aqueous myrrh extract demonstrated lower linear density, breaking tenacity, and elongation at the break.

Metal oxide nanomaterials like zinc oxide nanoparticles (ZnO NPs) are attracting a lot of attention in textiles as traditional material yarns can be efficiently decorated with these nanomaterials, bringing the necessary functionalities related to the wide optical band gap of ZnO [15,16]. Simple and sustainable ZnO deposition methods onto the textile are the key limiting factors for upscaling and mass production of multifunctional nanotextiles. Javed et al. [15] proposed a one-step hydrothermal method for deposition of ZnO from sodium hydroxide (NaOH) and zinc nitrate hexahydrate [Zn(NO_3_)_2_·6H_2_O] reactants. Optimized concentrations yielded 6.091 g/kg of Zn deposited on the cotton fabric. The amount of ZnO NPs on the surface of the fabric directly correlated with the antibacterial efficacy for *E. coli* and *S. aureus* bacteria and with catalytic activity confirmed using methyl orange. A UV protection factor reaching 130 for the sample containing the highest amount of Zn contents was demonstrated. Verbic et al. [16] proposed an entirely green in-situ synthesis of ZnO NPs on cotton fabric aiming for UV protective properties. Pomegranate peel extract was used as a reducing agent and wood ash extract was used as an alkali source for the formation of ZnO NPs from zinc acetate. It was obtained that synthesis methods that included continuous drying of the samples between immersion in the active solutions for synthesis were found to be the most suitable to deliver uniformly impregnated cotton fibers with numerous small ZnO wurtzite-structured crystals, and reached a UV protection factor of 154. 

A critical review by Repon et al. [17] indicated the importance of the recent advances in conductive textiles comprising conductive fibers, yarns, and fabrics, and the finished goods produced using them. These electrically conductive threads contain conductive substrates, metal wires, metallic yarns, nanomaterial additives, and intrinsically conductive polymers. Such smart textiles demonstrate numerous trade-offs in versatility, ergonomics, low energy utilization, integration, and heating properties.

## 3. Conclusions and Outlook

Comprehensive knowledge of the material synthesis and deposition methods used in nanotextiles, as well as an understanding of their physical, chemical, mechanical properties, biocompatibility, and associated environmental issues, requires a multidisciplinary approach. A critical mass of research has already been conducted, and nanomaterial-loaded textiles are eagerly waiting to be introduced into our daily life for taking care of harmful UV irradiation, fighting against bacterial infections, and gaining new functionalities through smart wearables. The ongoing transformation in wearable technology innovations is promising a similar paradigm shift in the textile industry to the invention of synthetic fibers.
